# Osteoarticular Manifestations of Prolidase Deficiency and Disability: Case Reports of Two Moroccan Sisters

**DOI:** 10.7759/cureus.17875

**Published:** 2021-09-10

**Authors:** Mouna Asly, Madiha Eljazouly

**Affiliations:** 1 Physical Medicine and Rehabilitation, Cheikh Khalifa International University Hospital, Mohamed VI University of Health Sciences (UM6SS), Casablanca, MAR; 2 Dermatology Unit, Cheikh Khalifa International University Hospital, Mohammed VI University of Health Sciences (UM6SS), Casablanca, MAR

**Keywords:** prolidase deficiency, osteoarticular manifestations, skin ulcers, skeletal deformities, musculoskeletal disability

## Abstract

Prolidase deficiency (PD) is a rare autosomal recessive disorder that has symptoms such as chronic skin ulcers, dysmorphic facies, cognitive retardation, hematological anomalies, splenomegaly, and chronic infections. Bone and joint abnormalities were referred occasionally and included the signs and symptoms of prolidase deficiency, but were not deeply investigated in PD patients. We report a case of two PD Moroccan sisters with osteoarticular deformities, some of them were never described before: toes deformities and equinovalgus with fusion and dislocation of a tarsal bone in radiography x-rays.

## Introduction

Prolidase Deficiency (PD) is a rare autosomal recessive disease associated with elevated plasma and urine imidodipeptides caused by mutations in the paroxysmal extreme pain disorder (PEPD) gene encoding for prolidase, an enzyme involved in the biosynthesis and degradation of collagen I, which is an important component of the organic extracellular matrix. The role of this matrix is to strengthen and support connective tissues, such as skin, bone, cartilage, tendons, and ligaments [1].

PD is defined by a protean clinical spectrum, characterized by lower limb ulcers, facial dysmorphisms, deafness, splenomegaly, cognitive impairment, and recurrent infections [2]. Bone and joint abnormalities were referred occasionally. This is a case study of two PD Moroccan sisters with skeletal deformities, causing significant disability. The consent was obtained from the patients for publication of the case and images.

## Case presentation

Case report one

Patient one, aged 39 years old, was admitted for lower limb deformities and gait disturbances. Since the age of five, the patient was kept under examination in the dermatology department for chronic ulcerations of the lower limbs, related to prolidase deficiency (PD). Anamnesis revealed first-degree parent consanguinity and a younger sister affected by the same disease.

**Figure 1:**
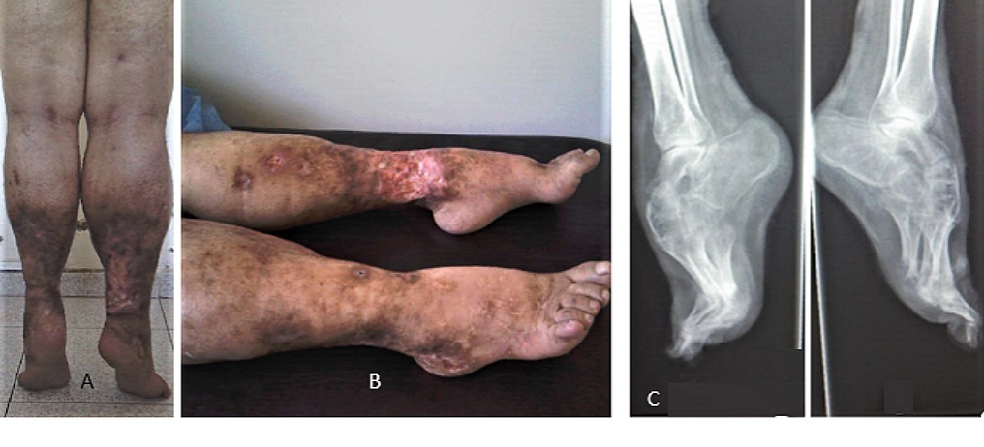
A-B: multiple geographic ulcers on the feet, ankles, and distal tibial areas on both legs, bilateral genu valgum, and bilateral irreducible equinus. C: Lateral foot x-ray: demineralization, and equinus, without joint involvement.

Clinical examination found multiple geographic ulcers with elevated borders on the feet, ankles, and distal tibial areas on both legs, bilateral genu valgum, and bilateral irreducible equinus. The plantigrade posture of the feet, being impossible, the patient was walking on tiptoe with a significant risk of falls (Figures 1A, 1B). Spine and upper limbs examination were normal. Knees and ankles x-rays showed significant demineralization and equinus, without joint involvement (Figure 1C). Orthopedic shoes, compensating the equinus and crutches have been proposed to improve balance and prevent falls.

Case report two

Patient two, aged 35 years old, is the younger sister of patient one and was also, diagnosed with PD when she was three years old.

**Figure 2:**
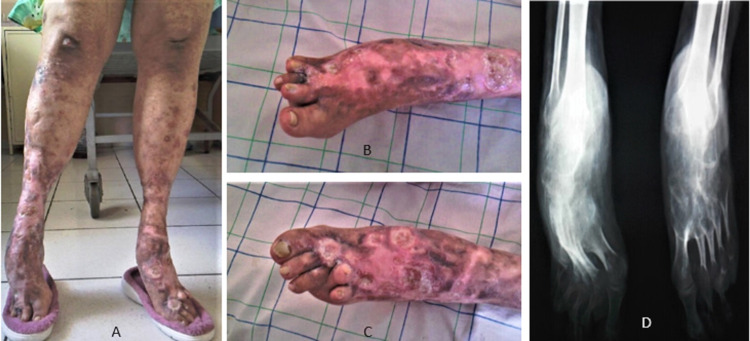
A: several ulcers, dry necrotic lesions, and retractile atrophic scars, bilateral genu valgum, irreducible equinus associated to varus of the right foot, and valgus of the left one. B-C: supra-adductus of fourth toes and of the right second toe. D: demineralization, bilateral genu valgum, fusion, and dislocation of the tarsal bones.

Clinical examination found on the lower limbs, several ulcers, dry necrotic lesions, and retractile atrophic scars, bilateral genu valgum, irreducible equinus associated to varus of the right foot and valgus of the left one (Figure 2A), and fixed toes deformities: supra-adductus of fourth toes and of the right second toe (Figures 2B, 2C). The patient is walking in a wheelchair. Spine and upper limbs examination were normal. Knees and ankles radiography x-rays showed diffuse demineralization, bilateral genu valgum, fusion, and dislocation of the tarsal bones (Figure 2D). 

## Discussion

PD is a rare autosomal recessive disorder. Since the first description of PD by Goodman in 1968, about 90 cases have been diagnosed all over the world. The incidence of PD is one to two per one million births, and it is more frequent in some populations, like the Druze and Arab Muslim minority in Israel [1].

Due to a severely reduced prolidase activity in PD, a large amount of proline remains in the form of imidodipeptides x-proline and x-hydroxyproline, which are excreted in the urine. Thus, the hallmark of PD is a massive imidopeptiduria associated with elevated proline or hydroxyproline containing dipeptides in plasma. Although there is considerable knowledge concerning the putative roles of the prolidase enzyme, the pathophysiology of PD is still not clearly understood, as there are marked phenotypic variability among affected individuals [1].

PD is a multisystem disorder, characterized by lower limb ulcers, facial dysmorphisms, deafness, splenomegaly, cognitive impairment, recurrent infections, and autoimmune manifestations [1,2]. Dermatological symptoms of PD are observed in the majority of patients, mainly in the form of leg ulcers which it is typically recurrent, severe, recalcitrant, and painful and usually appears in early childhood or in the teens. Telangiectasias, purpura, premature greying of the hair, photosensitivity, erythematous maculopapular rash, hypertrichosis, and dysmorphic facial features have also been described [3,4].

Bone and joint abnormalities were referred occasionally. The common manifestations reported are joint laxity, talipes equinovarus, genu valgum, short stature, osteoporosis, arachnodactyly, hip dislocation, and delayed bone age. An association between systemic lupus erythematosus and prolidase deficiency has been described: patients who were already known to have prolidase deficiency developed clinical and immunological abnormalities consistent with a diagnosis of systemic lupus erythematosus (SLE) and following treatment with oral prednisolone their clinical condition has improved [5-8].

Other deformities involving the spine include spina bifida of C3 and 13th thoracic vertebrae, a fusion of C2 and C3, short neck, and kyphoscoliosis [9,10]. Muscular abnormalities were also found such as wasting of the small muscles of the hand with simian creases and digital clubbing, in the presence and in the absence of pulmonary abnormalities [6, 11-12]. The diagnosis of prolidase deficiency is established by detection of either biallelic PEPD pathogenic variants or reduced prolidase enzyme activity in a patient who has characteristic clinical findings and imidodipeptiduria [13].

There is no curative treatment available for PD. It requires a multisystemic therapeutic approach to each symptom. Supportive treatment of skin and immunologic manifestations has been efficacious in some patients. Rehabilitative interventions are proposed as needed to address motor and cognitive impairments [1,13]. Genetic counseling is recommended if the PEPD pathogenic variants have been identified in the family. It allows carrier testing for at-risk relatives and prenatal testing for pregnancies at increased risk [13].

## Conclusions

This paper reports osteoarticular deformities, related to PD, not described before such as deformities of the toes and the equinovalgus with fusion and dislocation of the tarsal bone. The severity of motor disability induced by deformities confirms the importance of a multidisciplinary approach, when treating patients with rare syndromes such as PD, to improve their quality of life.
